# Human iN neuronal model of schizophrenia displays dysregulation of chromogranin B and related neuropeptide transmitter signatures

**DOI:** 10.1038/s41380-024-02422-x

**Published:** 2024-02-02

**Authors:** Sonia Podvin, Jeffrey Jones, Austin Kang, Ryan Goodman, Patrick Reed, Christopher B. Lietz, Joshua Then, Kelly C. Lee, Lisa T. Eyler, Dilip V. Jeste, Fred H. Gage, Vivian Hook

**Affiliations:** 1 Skaggs School of Pharmacy and Pharmaceutical Sciences, University of California, San Diego, La Jolla CA USA; 2 Salk Institute, San Diego, La Jolla CA USA; 3 Department of Psychiatry, University of California, San Diego, La Jolla CA USA; 4 Desert-Pacific Mental Illness Research Education and Clinical Center, VA San Diego Healthcare System, San Diego, CA 92161 USA; 5 Global Research Network on Social Determinants of Health, San Diego, La Jolla CA USA; 6 Department of Neurosciences, University of California, San Diego, La Jolla CA USA; 7 Department of Pharmacology, University of California, San Diego, La Jolla CA USA

**Keywords:** Neuroscience, Schizophrenia

## Abstract

Schizophrenia (SZ) is a serious mental illness and neuropsychiatric brain disorder with behavioral symptoms that include hallucinations, delusions, disorganized behavior, and cognitive impairment. Regulation of such behaviors requires utilization of neurotransmitters released to mediate cell-cell communication which are essential to brain functions in health and disease. We hypothesized that SZ may involve dysregulation of neurotransmitters secreted from neurons. To gain an understanding of human SZ, induced neurons (iNs) were derived from SZ patients and healthy control subjects to investigate peptide neurotransmitters, known as neuropeptides, which represent the major class of transmitters. The iNs were subjected to depolarization by high KCl in the culture medium and the secreted neuropeptides were identified and quantitated by nano-LC-MS/MS tandem mass spectrometry. Several neuropeptides were identified from schizophrenia patient-derived neurons, including chromogranin B (CHGB), neurotensin, and natriuretic peptide. Focusing on the main secreted CHGB neuropeptides, results revealed differences in SZ iNs compared to control iN neurons. Lower numbers of distinct CHGB peptides were found in the SZ secretion media compared to controls. Mapping of the peptides to the CHGB precursor revealed peptides unique to either SZ or control, and peptides common to both conditions. Also, the iNs secreted neuropeptides under both KCl and basal (no KCl) conditions. These findings are consistent with reports that chromogranin B levels are reduced in the cerebrospinal fluid and specific brain regions of SZ patients. These findings suggest that iNs derived from SZ patients can model the decreased CHGB neuropeptides observed in human SZ.

## Introduction

Schizophrenia (SZ) is a debilitating mental illness and neuropsychiatric brain disorder which results in hallucinations, delusions, disorganized thoughts and behavior, and cognitive impairment [1–4]. The fundamental basis of brain functions in health and disease is the reliance on neurotransmitter molecules that are essential for cell-cell communication among the brain regions responsible for the spectrum of behavioral deficits in SZ.

Investigations in the field have determined that SZ is associated with elevated levels of the catecholamines dopamine, norepinephrine, and epinephrine [5–7]. This finding is further supported by the action of typical antipsychotics, which exert their therapeutic effects via dopamine receptor antagonism [8–10]. However, antipsychotics are unable to improve the cognitive dysfunction of SZ patients, which may be hypothesized to result from abnormalities in other neurotransmitter systems.

Peptide neurotransmitters, known as neuropeptides, comprise the largest portion of transmitters in the nervous system [11–15]. Active neuropeptides are derived from proneuropeptide precursors by proteolytic processing [11, 12]. Profiling of neuropeptides by mass spectrometry peptidomics has revealed the diversity of neuropeptides; there are hundreds to likely thousands of distinct peptides. The primary amino acid sequence of each neuropeptide defines its unique biological activity, which is mediated via peptidergic receptors. Neurotransmission by neuropeptide signaling is achieved together with the classical small molecule neurotransmitters [16, 17]. These small molecule transmitters include acetylcholine, adenosine, anandamide, aspartate, dopamine, epinephrine, GABA, glutamate, glycine, histamine, melatonin, norepinephrine, serine, and serotonin.

Based on the prevalence of the neuropeptides utilized for synaptic neurotransmitter signaling, this study hypothesized that alterations in neuropeptide signatures might occur in human SZ neurons compared to those from healthy controls. To assess this hypothesis, we utilized patient-derived induced neurons (iNs) as a human cellular model of SZ. The iNs are generated by transforming patient fibroblasts into iNs using a programming process that retains the aging phenotype, based on transcriptomics signatures [18, 19]. Therefore, the advantage of the iNs is that these neuronal phenotypes represent the adult age range of the neurons involved in SZ, rather than neurons at the fetal developmental stage that are represented by induced pluripotent stem cell (iPSC) neurons.

In this study, the iNs derived from SZ patients and control subjects (of similar age range) were utilized as a model of neurotransmitter secretion occurring during high KCl depolarization.. The spectrum of secreted neuropeptides was analyzed by neuropeptidomics mass spectrometry to identify and quantitate the endogenous neuropeptides. Results showed that neuropeptide signatures in SZ were dysregulated compared to healthy controls. Notably, distinct chromogranin B-derived (CHGB-derived) neuropeptides were secreted from the SZ neurons compared to those from healthy controls, and fewer CHGB-derived peptides were observed in the SZ group compared to the controls. Furthermore, the iNs secreted neuropeptides under both KCl and basal (no KCl) conditions. These findings are consistent with reports of reduced CHGB neuropeptides in SZ brains [20, 21], but prior studies had not identified the distinct CHGB peptides unique to SZ that were observed in this project. These findings suggest that iNs derived from SZ patients can model the decreased CHGB-derived neuropeptides observed in human SZ.

## Materials and methods

### Preparation of iNs from human dermal fibroblasts of SZ and healthy control skin biopsies

**Fig. 1:**
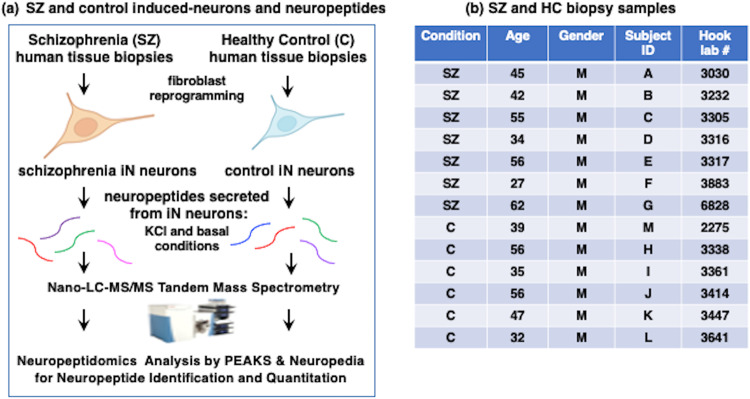
Strategy for neuropeptide studies of iNs derived from schizophrenia (SZ) and healthy control (HC) subjects. **a** Induced neurons (Ins) derived from tissue biopsies from SZ and HC subjects. The iNs were generated by direct reprogramming of fibroblasts obtained from SZ and HC tissue biopsies. Neurons were subjected to KCl (50 mM) stimulation of secretion and the media was collected for neuropeptidomics analysis achieved by nano-LC-MS/MS tandem mass spectrometry and bioinformatics using PEAKS and NeuroPedia for identification and quantitation of neuropeptides. **b** SZ and HC biopsy samples. Individual SZ and HC biopsy samples are listed by condition of SZ or HC, age, and gender. The sample sizes were selected based on power analysis to characterize neuropeptide identities and quantities with significance. Note that the predicted effect was not known at the beginning of this project, and effects of only in SZ or only in HC were observed with SZ (*n* = 7) and HC (*n* = 6) numbers of patient-derived cell lines in this study.

Direct reprogramming of human neurons, iNs, from human tissue biopsies was conducted as we have described previously [18, 19]. We have shown that the iNs retain the original age phenotype of the parent human cell source based on transcriptomics signatures (18, 19). For preparation of iNs, dermal fibroblasts derived from skin punch biopsies were obtained from SZ patients and age-matched healthy control subjects according to protocols approved by the Institutional Review Board at UC San Diego. Biopsy information is provided in Fig. 1a. Skin punch biopsies were acquired from the deltoid region and primary fibroblasts were cultured using methods described previously [18, 22, 23]. Passage 2 to 3 primary fibroblasts were grown at 37 °C and 5% CO_2_ and cultured in DMEM with 15% fetal bovine serum (FBS) and 0.1% non-essential amino acids (NEAA; Life Technologies). For the iN conversion process, fibroblasts were transduced with lentiviral particles XTP-Ngn2:2A:Ascl1 (with EtO vector) for expression of the proneural transcription factors Ngn2 (neurogenin-2) and Ascl1 (BHLH family of transcription factors) used to induce the fibroblasts into neurons, conducted as previously described [18]. Viral transduction was conducted for 2 days to induce differentiation into the pre-induced neuron (pre-iN) fibroblast phenotype, followed by media containing 10 μg/ml puromycin for selection of transgene-expressing cells. For neural differentiation into iNs, cells were incubated with conversion media containing 50% DMEM, 50% Neurobasal media, supplemented with N2, B27 (Gibco), doxycycline (2 μg/ml, Sigma Aldrich), Laminin (1 μg/ml, Life Technologies), dibutyryl cyclic-AMP (500 μg/ml, Sigma Aldrich), human recombinant Noggin (150 ng/ml, Preprotech), LDN-193189 (0.5 μM, Cayman Chemical Co) and A83-1 (0.5 μM; Stemgent), CHIR99021 (3 μM, LC Laboratories), Forskolin (5 μM, LC Laboratories) and SB-431542 (10 μM, Cayman Chemicals). Neural differentiation proceeded for 3 weeks, with media changed every third day. Finally, iNs were matured for 1 week in the presence of 50% DMEM, 50%F12/Neurobasal-based neural maturation media (NM) containing N2, B27, GDNF, BDNF (both 20 ng/ml, R&D), dibutyryl cyclic-AMP (500 μg/ml, Sigma Aldrich), doxycycline (2 μg/ml, Sigma-Aldrich) and laminin (1 μg/ml, Life Technologies).

The iNs were characterized by the neuron-specific markers MAP2 (microtubule-associated protein 2) [24, 25] and beta-III tubulin [26] by immunofluorescent imaging using antibodies to TUJ-1 (beta-III tubuilin, Covance, #A488-435L, rabbit, at 1:2500 dilution) and antibody to MAP2 (Sigma #M2320, mouse, at 1:200 dilution), conducted as we previously described [25]. Briefly, cells were fixed in 4% PFA at room temperature for 15 min. Cells were washed with PBS 1x and then blocked and permeabilized in a single step consisting of 0.2% Triton and 4% horse serum for 1 hour at room temperature. The solution was aspirated and replaced with primary antibodies in a 1:1 dilution of blocking solution and left in 4 °C overnight. PBS washes [3x] were applied the next day and secondary antibodies were added, diluted in PBS. Secondary antibodies used were Alexafluor 546 donkey anti-rabbit (Invitrogen #A10040, 1:1000) and Alexafluor 488 donkey anti-mouse (Invitrogen #A-21202, 1:1000) at room temperature for 1 h. Finally, DAPI (Sigman #D9542) was applied at 1:300 for 10 min and washed twice. Slides were imaged on a Zeiss Observer.Z1 with a 20x air objective. Zen 2 Pro software was used to capture images. Individual channels were separated by Fiji. In addition, iN cultures were also assessed for immunofluorescent staining with glia cell markers consisting of GFAP (Agilent/Dako #Z033429-2, 1:5000), S100B (Thermo #PIMA512969, 1:200), and Iba 1 (Fugifilm/Wako Chemicals #NC9288364, 1:1000).

Assessment of the iN neurons in our prior study assessed morphological features, neuronal markers, electrophysiology, and transcriptomics. This extensive characterization showed that the iN neurons displayed mature neuronal morphology, synapsin-I staining at neuritic intersections, and evoked and spontaneous action potential firing [18]. The action potential firing is consistent with activity-dependent secretion of neurotransmitters, representing neuropeptides evaluated in our study. Furthermore, the majority of the iN neurons showed a glutamatergic fate (about 80%) and a modest portion of neurons were GABA-positive (15–20%) [18]. Transcriptomics analysis indicated neuronal functions indicated by enrichment for GO terms of synaptic transmission, neurotransmitter transport, generation of neurons, neuron differentiation, regulation of synapse organization, and neurogenesis. These data demonstrate the in-depth characterization of the neuronal properties of the iN neurons.

### Neuropeptide secretion from iNs treated with high KCl

Three cell culture replicates from each subject were utilized in KCl treatment (50 mM), which stimulates neurotransmitter release. Neural maturation media was removed and cells were washed twice with phosphate buffered saline (PBS). Cells were equilibrated in standard release media for 15 minutes at 37 °C (SRM: 118 mM NaCl, 4.6 mM KCl, 10 mM D-glucose, 25 mM HEPES pH 7.4, 2.2 mM CaCl_2_ and 1.2 mM MgSO_4_). Next, cells were incubated with SRM containing 50 mM KCl for 30 min, or SRM without KCl that represented basal secretion. Note that protease inhibitors are not present in the SRM media since it has been shown that released secretory proteases can function extracellularly at neutral pH for processing and production of neuropeptides [12]. After the SRM/50 mM KCl treatment, media was collected and centrifuged at 500 × *g* for 3 min to pellet detached cells, and supernatant was transferred to a clean collection tube and flash-frozen in liquid N_2_.

### Extraction of peptides from culture media

Peptides were precipitated by acid peptide extraction and solid phase extraction as described previously [12]. Briefly, secretion samples were acidified 23:1 v/v with cold 1 M HCl to lower pH < 3. Samples were vortexed and incubated on ice and then centrifuged at 14,000 × *g* for 30 min at 4 °C to pellet proteins. Supernatant-containing peptides were reserved. Samples were further desalted and purified by C18 solid phase extraction. Following elution, samples were dried in a speed-vac and resuspended in 2% acetonitrile, 0.1% trifluoroacetic acid for nanoLC-MS/MS analysis.

### Data-dependent peptidomics conducted by nano-LC-MS/MS tandem mass spectrometry

Analyses were performed using a Dionex UltiMate 3000 nano liquid chromatography system and a Q-exactive mass spectrometer (Thermo Scientific). For nano-LC-MS/MS, samples were each injected twice at 1 μl volume and separated by reverse phase chromatography on a 1.7 μm bridged-ethylene hybrid C18 bead column (75 μm inner diameter, 25 cm length, heated to 65 °C) at a flow rate of 300 nl/min. Samples were eluted over a continuous gradient of 5% acetonitrile, 0.1% formic acid to 40% acetonitrile, 0.1% formic acid for 40 min, followed by elution in 95% acetonitrile, 0.1% formic acid for 10 min. MS1 scans were acquired over a range of 310–1250 m/z in profile mode with resolution of 70,000 at 200 m/z, 150 ms maximum inject time and AGC target of 3 ×10^6^. MS/MS were acquired in data-dependent mode with HCD fragmentation of 28 (N)CE, resolution of 17,500 at 2.0 m/z, ACG target of 2 ×10^5^, and first mass at m/z of 150.

### Bioinformatics of peptidomics data for neuropeptide identification and quantitation

A targeted bioinformatic search for neuropeptides utilized the protein sequences for all known human proneuropeptide precursors [12, 27] to query spectra using PEAKs v. 8.5 (Bioinformatics Solutions, Inc., Waterloo, ON, Canada) for peptide sequence identification. Data filtration for false discovery rate of 0.3% was achieved by decoy-fusion spectrum library search, with MS1 mass error tolerance of 20.0 ppm and fragment mass error tolerance of 0.01 Da. Post-translational modifications included in the search were methionine oxidation, pyro-glutamate, N-term acetylation and amidation. Quantification of identified peptides was determined by label-free quantification (LFQ) by PEAKs v. 8.5. Quality parameters for quantification were restricted to peptide abundance of >1 ×10^4^ and quality >0.3. The extracted ion chromatographs (XIC) of MS2 peaks were converted to area under the curve (AUC). Inter-replicate reliability of XIC data were restricted by peptide features of retention time within 3 min, and precursor features including mass, peak height, intensity and isotope pattern. A peptide was considered identified and quantified in a subject if present in at least 1 of 2 technical replicates, present in at least 2 out of 3 cell culture replicates, present in the SZ group if present in at least 5 of 7 subjects, and present in the control group in at least 4 of 6 subjects. Label-free quantitation (LFQ) of peptides was expressed as mean ± s.e.m; student’s two-tailed t-test of <0.05 determined significance levels between SZ and control groups. Group variance values were found to be similar.

## Results

### Strategy to assess neuropeptide signatures secreted from human SZ and healthy control iNs by peptidomics mass spectrometry

Human iNs were studied as models of human SZ and control neurons (Fig. 1a). Patient-derived iNs were generated by obtaining skin biopsies from SZ (7 subjects) and healthy control (6 subjects) patients of a similar range in ages consisting of 30–60 years (Fig. 1b). This study utilized all male subjects to reduce variability since sex differences in SZ features have been observed [28]. After reprogramming biopsy-derived fibroblasts into iNs, the neurons were treated with high KCl (50 mM), a model for depolarization induction of neurotransmitter secretion into the media [29]. Basal secretion was assessed without KCl. Peptides in the collected media were isolated by acid-MeOH extraction to precipitate proteins and the soluble peptides were analyzed by neuropeptidomics mass spectrometry combined with bioinformatics using PEAKS and NeuroPedia for identification and label-free quantitation (LFQ). Quantitative neuropeptide data were assessed for differences and similarities between the SZ and control groups.

### iN cells display neuron-specific markers

**Fig. 2:**
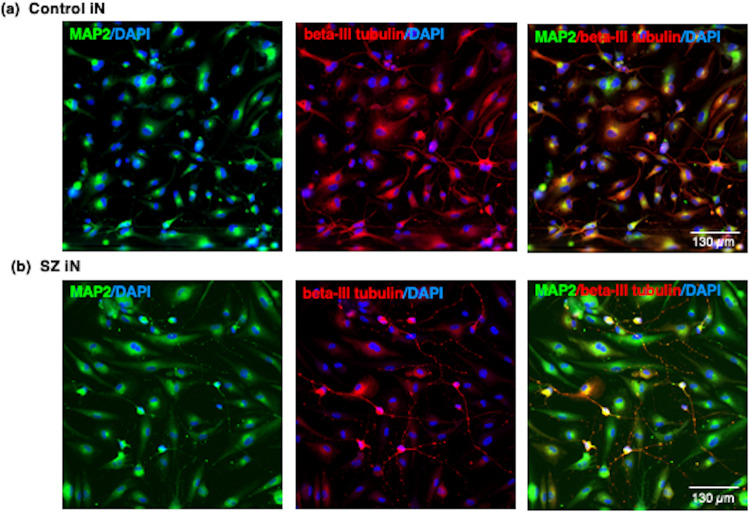
Induced neurons (iN) display neuron-specific markers of MAP2 and beta-III-tubulin. The patient-derived iN cells derived from control (panel **a**) and SZ (panel **b**) tissue biopsies were subjected to immunofluorescent imaging with MAP2 (FITC-label, green fluorescence) and beta-III tubulin (TRITC-label, red fluorescence) neuron-specific markers [24–26]. Cell nuclei are indicated by DAPI staining (blue fluorescence). MAP2 and beta-III tubulin are shown separately and as merged images.

The neuron-specific markers MAP2 and beta-III tubulin were utilized to characterize neurons of the iN preparations derived from tissue biopsies from control and SZ subjects. Control and SZ iNs all displayed MAP2 (microtubule-associated protein 2), a marker of differentiated neurons (Fig. 2). The iNs also showed beta-III tubulin immunofluorescent staining that represents neuritic extensions. The MAP2 and beta-III tubulin immunofluorescence was observed in the cells outside of nuclei (illustrated by DAPI immunofluorescence). Further, there was an absence of of glia cell marker expression consisting of GFAP (glia fibrillary acidic protein) and S100B markers for astrocytes, and the Iba1 marker for microglia cells. These findings indicate that a pure preparation of 100% neuronal cells was generated by the iN protocol.

### Distinct and shared neuropeptides secreted from SZ and control iNs treated with KCl illustrate dysregulation of chromogranin B neuropeptides

**Fig. 3:**
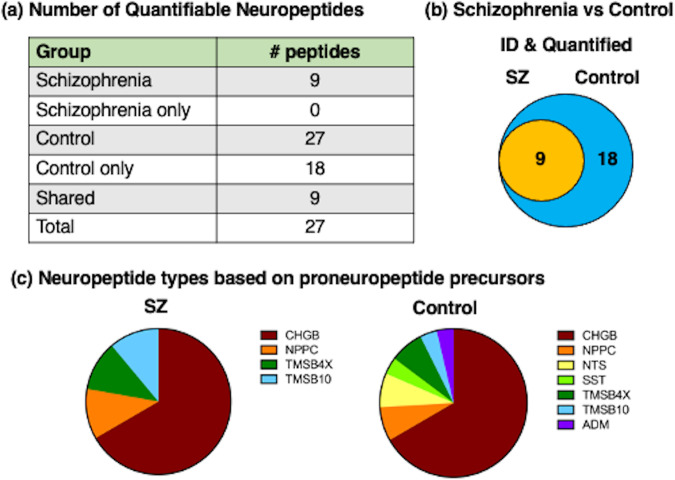
Neuropeptidomics analysis of secreted neuropeptide signatures from SZ iNs and control iNs treated with high KCl. **a** Neuropeptide counts from SZ and HC iN neuronal secretion media. The number of neuropeptides identified and quantitated from the SZ and HC groups are shown. **b** Venn diagram of SZ compared to HC neuropeptides. The venn diagram shows the unique neuropeptides present in only SZ or only HC groups and the neuropeptides that are shared by both groups. **c** Proneuropeptides of identified neuropeptides. Proneuropeptide precursors are viewed according to the number of neuropeptides found from each precursor.

KCl (50 mM) was used as a model of depolarization-induced neurotransmitter secretion that is utilized for neuronal release of neurotransmitters [17, 29]. Both differences and similarities in the neuropeptides secreted from SZ iNs compared to those of healthy controls were observed (Fig. 3a, b). A total of 27 neuropeptides were identified and quantitated in both groups. All of the 9 identified SZ neuropeptides were present in the control group. However, 18 of the control group neuropeptides were not present in the SZ group. These data show that the SZ group contained one-third of the neuropeptides present in the control group, indicating that two-thirds of the normal signature of secreted neuropeptides were lost in the SZ group.

Neuropeptides are generated from proneuropeptide precursors by proteolysis, and each proneuropeptide can generate several distinct neuropeptides. The numbers of neuropeptides derived from each proneuropeptide precursor found in the SZ and HC groups are illustrated by Venn diagrams (Fig. 3c). The majority of the SZ neuropeptides were derived from the chromogranin B (CHGB) proneuropeptide precursor (Fig. 3c). In addition, several of the SZ neuropeptides consisted of the C-type natriuretic peptide (NPPC), thymosin β-4 (TMSB4X), and thymosin β-10 (TMSB10) types of neuropeptides. The control iNs secreted a large number of CHGB-derived neuropeptides (numbering 12), combined with the neuropeptides of NPPC, TMSB4X, TMSB10, neurotensin (NTS), somatostatin (SST), and adrenomedullin (ADM) (Fig. 3c). The numbers of neuropeptides derived from each of these proneuropeptides in the SZ and control groups are summarized in Table 1.

**Table 1:** Neuropeptides secreted from schizophrenia (SZ) and control (C) induced neurons (iNs) in the presence of KCl.

Proneuropeptide	SZ	SZ only	C	C only	Shared	Total
CHGB	6	0	18	12	6	18
NPPC	2	0	2	1	1	2
NTS	0	0	2	2	0	2
SST	0	0	1	1	0	1
TMSB4X	1	0	2	1	1	2
TMSB10	1	0	1	0	1	1
ADM	0	0	1	1	0	1

The numbers of neuropeptides in SZ and C secretion media from iN cells treated with KCl (50 mM) derived from the indicated proneuropeptides are indicated. Neuropeptides were identified and quantitated by nano-LC-MS/MS tandem mass spectrometry and PEAKs bioinformatics as described in the methods. Comparison of the SZ and C groups show neuropeptides found in only the C group combined with those found in both SZ and C groups (shared).

### Distinct CHGB-derived peptides secreted from SZ compared to control iNs

**Fig. 4:**
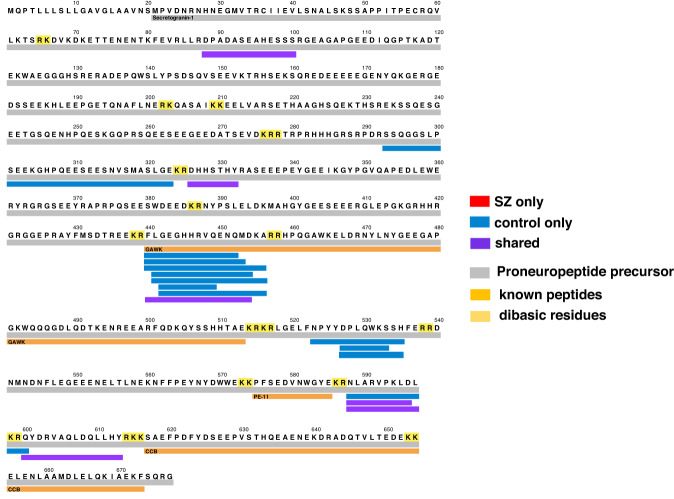
Peptide mapping reveals differences in chromogranin B (CHGB)-derived neuropeptides secreted from SZ and control iNs treated with high KCl. Neuropeptides derived from the CHGB proneuropeptide were mapped according to those present only in the control group, only in the SZ group, or shared by both groups. Neuropeptides were identified and quantitated in secretion media obtained from iNs in the condition of high KCl depolarization.

Peptide mapping of neuropeptides (secreted during KCl treatment) to the parent CHGB proneuropeptide precursor illustrated several distinct CHGB-derived neuropeptides (12 neuropeptides) that were absent in the SZ group but were present in the control group (Fig. 4). Thus, the control group possessed unique CHGB-derived neuropeptides that were not observed in the SZ group. Only 6 peptides were present in both SZ and control groups, indicating that the majority of CHGB-derived neuropeptides differed between the two groups. Analysis of CHGB-derived neuropeptides among each of the SZ and control iN neuronal lines derived from individuals showed major similarities and some differences within each group (Table 2). These data showed that CHGB neuropeptides were the major group of dysregulated neuropeptides secreted from the SZ iNs compared to controls.

**Table 2:**
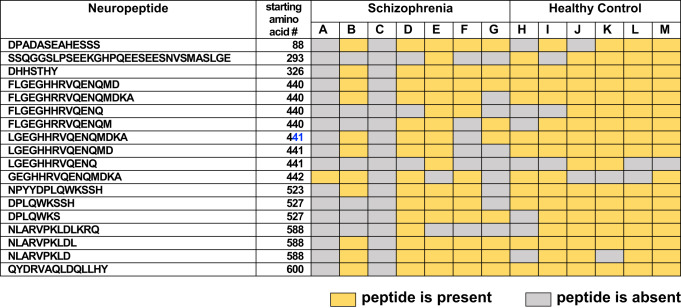
CHGB-derived neuropeptides present or absent in schizophrenia or control In secretion media of iNs treated with high KCl .

The presence or absence of CHGB-derived neuropeptides secreted from schizophrenia or control iN cells, in the presence of high KCl (50 mM), derived from subjects A-M are illustrated.

**Fig. 5:**
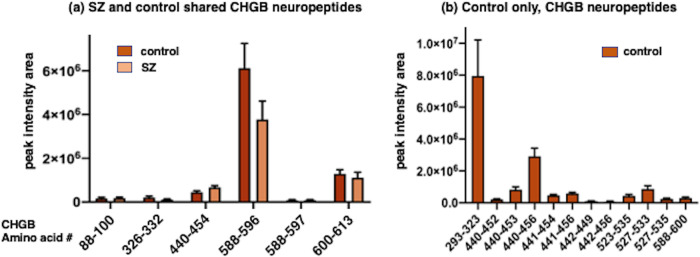
Quantitation of CHGB-derived neuropeptides present in both SZ and control iNs, and present in only control iNs. **a** CHGB-derived neuropeptides present in both SZ and control iNs. Neuropeptides derived from the CHGB proneuropeptide were identified and quantitated in KCl secretion media of both SZ and control iNs. Relative neuropeptide levels are indicated as peak intensity area from LC-MS/MS. Neuroepeptide levels for each condition are shown as means ± s.e.m (*n* = 7 for the SZ group, and *n* = 6 for the control group). **b** CHGB-derived neuropeptides present in only control iNs. CHGB neuropeptides observed in only the control iN group were quantitated as described in the methods. Data are shown as the mean ± s.e.m. (*n* = 6 iN control cell lines).

The quantities of CHGB neuropeptides secreted from both SZ and control iNs (in the presence of KCl) show differing levels of such secreted peptides (Fig. 5a). The CHGB 588–596 neuropeptide was secreted from SZ and control groups at the highest levels, CHGB 440–454 and CHGB 600–613 were secreted at more modest levels, and low levels CHGB peptides 88–100, 326–332, and 588–597 were secreted. Under the KCl secretory condition, the SZ and control groups secreted similar levels of each CHGB peptide. The control group secreted 12 CHGB peptides that were not observed in the SZ group (Fig. 5b); these control CHGB peptides were secreted at varying high to lower levels (Fig. 5b).

### Differential NPPC, NTS, and SST neuropeptide profiles secreted from SZ compared HC iNs during KCl treatment

**Fig. 6:**
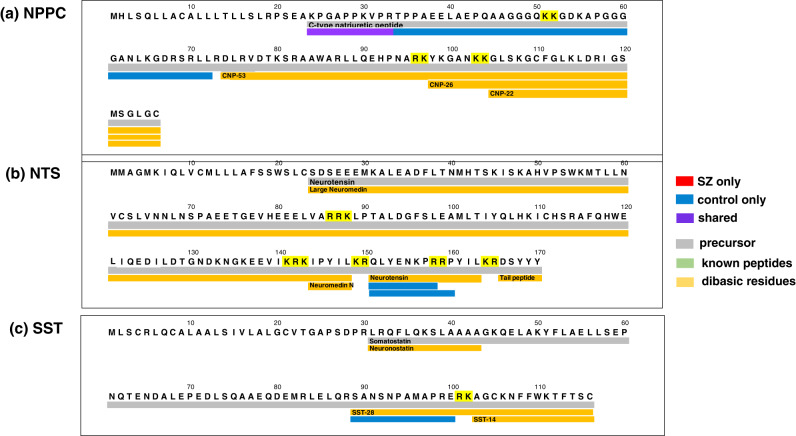
Natriuretic, neurotensin, and somatostatin neuropeptides mapped to proneuropeptide precursors. Neuropeptides identified in these studies of SZ and control iNs (under high KCl condition) were mapped to each of their proneuropeptide precursors according to peptides present only in the control group, only in the SZ group, or shared by both groups. Neuropeptide mapping is illustrated in panels **a**–**c** showing (**a**) C-type natriuretic, (**b**) neurotensin, and (**c**) somatostatin.

**Fig. 7:**
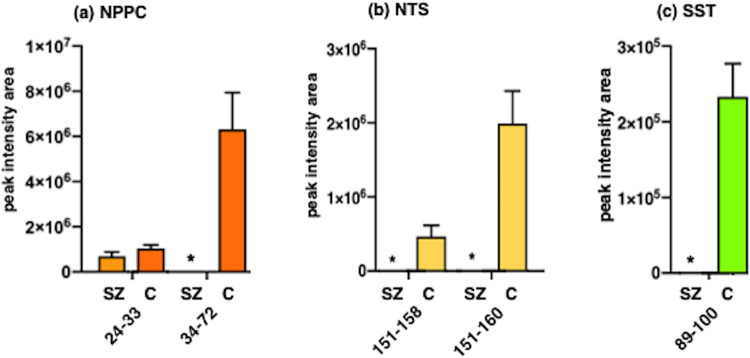
Quantitative comparison of NPCC, NTS, and SST neuropeptides secreted from SZ and control iNs in the presence of high KCl. NPCC, NTS, and SST neuropeptides secreted from SZ and control (C) iNs are illustrated in panels **a**, **b**, and **c**, respectively. Quantitation of neuropeptides are shown as mean +/- s.e.m. (*n* = 7 for SZ iN cell lines, and *n* = 6 for control iN cell lines).

Other identified neuropeptides were found largely in only the control group or were shared by both control and SZ groups (Fig. 6 and Table 1); these data indicate the loss of such control neuropeptides from the SZ iNs. Specifically, for the C-type natriuretic (NPPC) neuropeptides, one was present in only the control group and another neuropeptide was shared by the SZ and control groups (Fig. 6a). Two identified neurotensin (NTS) neuropeptides were present in only the control group (Fig. 6b). One somatostatin (SST) neuropeptide was observed in only the control group (Fig. 6c). The neuropeptide peak intensity values were quantitated for the NPCC, NTS, and SST neuropeptides observed in only the control group and not in the SZ group (Fig. 7).

For thymosin β-4 neuropeptides (TMSB4X), one was found only in control iNs and one peptide was shared by both SZ and control groups (Table 3). The thymosin β-10 neuropeptide (TMSB10) was shared by both groups (Table 3). One adrenomedullin neuropeptide (ADM) was found in only the control iNs (Table 3).

**Table 3:** Neuropeptides secreted under KCl and basal conditions from schizophrenia (SZ) and control (C) iNs.

Proneuropeptide	Peptide (aa #s)	SZ	SZ	C	C
		KCl	basal	KCl	basal
ADM	163–183	−	−	+	+
CHGB	88–100	+	+	+	+
	293–323	−	−	+	+
	326–332	+	+	+	+
	440–452	−	−	+	−
	440–453	−	−	+	−
	440–454	+	−	+	+
	440–456	−	−	+	+
	441–454	−	−	+	−
	441–456	−	−	+	+
	442–452	−	−	+	−
	442–456	−	−	+	−
	523–535	−	−	+	−
	527–533	−	−	+	+
	527–535	−	−	+	−
	588–596	+	+	+	+
	588–597	+	+	+	+
	588–600	−	−	+	−
	600–613	+	+	+	+
NPPC	24–33	+	+	+	+
	24–72	−	−	−	−
	34–72	−	−	+	+
	46–69	−	−	−	−
NTS	151–158	−	−	+	−
	151–160	−	−	+	+
SST	89–100	−	−	+	+
TMSB10	2–44	+	+	+	+
TMSB4X	2–19	−	−	+	+
	2–44	+	+	+	+

Neuropeptides in the media of basal and KCl secretion conditions, released by SZ or C iNs, were identified and quantitated as described in the methods. Proneuropeptide-derived neuropeptides are indicated.

Overall, there was a loss of chromogranin B and other selected neuropeptides secreted from the SZ iNs compared to control iNs during secretion in the presence of high KCl depolarization.

### Comparison of KCl and basal secretion of neuropeptides from SZ and control induced neurons (iNs)

**Fig. 8:**
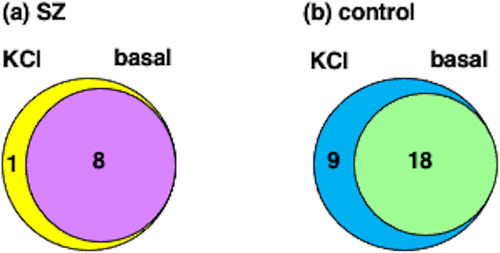
Neuropeptides secreted under high KCl compared to basal conditions from SZ and control iNs. **a** SZ iN neuropeptides secreted under KCl and basal conditions. The venn diagram illustrates neuropeptides secreted under KCl and basal conditions from SZ iNs. **b** Control iN neuropeptides secreted under KCl and basal conditions. Comparison of neuropeptides secreted under KCl and basal conditions from control iNs are shown.

Assessment of neuropeptides secreted under KCl compared to basal (no KCl) conditions showed that control iN neurons displayed KCl-stimulated secretion of 9 neuropeptides that differed from neuropeptides secreted in the basal condition which consisted of 18 peptides (Fig. 8). These results indicate that the healthy control iNs released 9 neuropeptides in a KCl activity-dependent manner out of a total of 27 secreted neuropeptides. The SZ iN cells, however, displayed primarily only basal secreted neuropeptides consisting of 8 peptides whereas only 1 peptide was uniquely secreted under the KCl condition (Fig. 8).

**Fig. 9:**
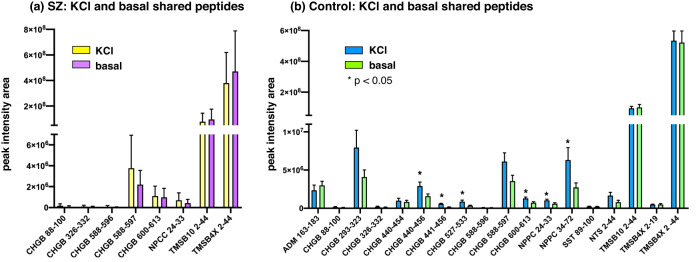
Neuropeptides secreted under KCl and basal conditions from SZ and control iNs. **a** SZ secreted neuropeptides. Neuropeptides secreted under both KCl and basal conditions from SZ iNs were quantitatively compared. Results are shown as means ± s.e.m. (*n* = 7 for SZ iNs). **b** Control secreted neuropeptides. Neuropeptides secreted under both KCl and basal conditions from control iNs were quantitatively compared. Results are shown as means ± s.e.m. (*n* = 6 for control iNs).

Quantitation of neuropeptides secreted in KCl and basal conditions from SZ and control iNs was assessed (Fig. 9). In the SZ group (Fig. 9a), relatively modest to high amounts of CHGB 588–597, TMSB10 2–44, and TMSBAX 2–44 neuropeptides were secreted under both KCl and basal conditions. Low levels of CHGB 600–613 and NPCC 24–33 were observed, combined with lower amounts of the CHGB 88–100, 326–332, and 588–596 neuropeptides. For control iN cells (Fig. 9b), ranges of high, moderate and low levels of neuropeptides were secreted in both KCl and basal conditions. High levels of TMSB10 2–44 and TMSBAX 2–44 were released, combined with moderate levels of CHGB 293–323, CHGB 588–597, and NPPC 34–72, as well as lower levels of the other 13 released neuropeptides; amounts secreted under KCl and basal conditions were similar for these neuropeptides.

Comparison of KCl and basal secretion conditions (Fig. 9) showed that control iNs displayed significant KCl-stimulated release of CHGB peptides 440–456, 441–456, 527–533, and 600–613 combined with the NPPC 24–33 and 34–72 neuropeptides. Several other neuropeptides did not show significant elevated release with KCl in the control group. SZ iNs did not show significant KCl-stimulated release of several neuropeptides compared to the basal condition. It is known that human iNs are electrically active [30, 31] which may preclude further activation of secretion by KCl.

### Basal secretion of neuropeptides in SZ compared to control iNs

**Fig. 10:**
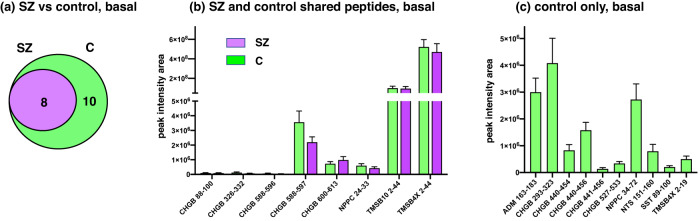
Basal secretion of neuropeptides from SZ and control iNs. **a** SZ compared to control iNs for basal secretion of neuropeptides. Comparison of neuropeptides secreted under basal conditions from SZ and control (C) iNs is illustrated by a Venn diagram. **b** Basal secretion of neuropeptides from both SZ and control iNs. Neuropeptides secreted from both SZ and control iNs under basal conditions were quantitatively compared, shown as means ± s.e.m. (*n* = 7 for SZ iNs, and *n* = 6 for control iNs). **c** Neuropeptides secreted only from control iNs by basal secretion. Neuropeptides secreted from only the control iNs under basal conditions are quantitatively illustrated (*n* = 6, control iNs).

Analysis of neuropeptides secreted under the basal condition (no KCl) showed that the 8 SZ neuropeptides were also secreted from control iN cells (Fig. 10a). Notably, 10 distinct control neuropeptides were secreted from only control iNs and not from the SZ iN neurons (Fig. 9a). For peptides basally secreted from both SZ and control neurons, high levels of TMSB10 2–44 and TMSBAX 2–44 peptides were released, moderate levels of CHGB 588–597, CHGB 600–613, with NPPC 24–33 were secreted, and lower levels of CHGB 88–100, 326–332, and 588–596 were released (Fig. 10b). For basal secretion of neuropeptides from only control iN neurons, high to moderate levels of neuropeptides were released which consisted of 5 CHGB peptides, one peptide each derived from the ADM, NPPC, NTS, SST, and TMSBAX proneuropeptides (Fig. 10c).

These data demonstrate secretion of neuropeptides in both conditions of KCl treatment and basal (no KCl) conditions. The majority of these secreted neuropeptides were derived from the CHGB proneuropeptide.

## Discussion

This study assessed the hypothesis that neuropeptide transmitters may be dysregulated and were, thus, modeled in SZ-derived iNs compared to healthy control iNs generated from human SZ and control tissue biopsies. The condition of high KCl (50 mM) was used to model neurotransmitter release under depolarizing conditions that induce secretion of transmitters [17, 29]. Neuropeptidomics analysis revealed losses of particular neuropeptides secreted from the SZ iNs compared to control iNs. Chromogranin B (CHGB) neuropeptides were the major group of secreted neuropeptides. Mapping of CHGB neuropeptides to its proneuropeptide precursor indicated that the SZ iNs displayed an absence of one-third of the neuropeptides present in the control iNs. Other secreted neuropeptides from the iNs were also observed which consisted of C-type natriuretic peptide (NPPC), neurotensin (NTS), somatostatin (SST), thymosin β-4 (TMSB4X), thymosin β−10 (TMSB10), and adrenomedullin (ADM). A large portion of these neuropeptides were absent in the SZ group or present in both groups. These findings suggest a loss of selected neuropeptides in the SZ iNs, with CHGB neuropeptides representing the major group of neuropeptide dysfunction modeled by the SZ iNs.

Alterations in the neuropeptide signatures derived from the proneuropeptide may occur through differential proteolytic processing of precursors or by degradative mechanisms. It will be of interest in future studies to evaluate predicted alterations in protease systems utilized by the SZ iNs compared to the control iNs for neuropeptide biosynthesis.

Comparison of neuropeptides secreted under KCl compared to basal (no KCl) conditions was assessed. Control iN neurons displayed activity-dependent secretion of neuropeptides, consistent with the knowledge that neurotransmitter secretion occurs under depolarizing conditions. Interestingly, the SZ iN neurons displayed very few KCl-stimulated neuropeptides, which numbered one neuropeptide. It is known that human iNs are electrically active [30, 31] which may preclude further activation of secretion by KCl. It will be of interest in future studies to assess the status of the electrical network activity in SZ and control iNs.

The findings of this study are consistent with studies of SZ patients indicating that chromogranin B levels are reduced in the cerebrospinal fluid and brain [20, 21]. Genetic polymorphisms in the CHGB gene have been identified in SZ human patients [32, 33], but such CHGB polymorphisms were not found in the neuropeptides secreted from iNs derived from SZ subjects in this study. It may be of interest in future studies to examine iNs derived from SZ patients with known polymorphisms in the CHGB gene to observe the consequences in CHGB neuropeptides. Furthermore, the lack of neurotensin and somatostatin types of neuropeptides in SZ iNs parallels findings in the literature demonstrating loss of such neuropeptides in SZ patients [34–37].

SZ involves deficits in memory and other cognitive functions that are not improved by antipsychotic drugs [38, 39]. Thus, while the SZ patients were administered antipsychotic drugs [40], this iN study can reveal changes in neuropeptides known to participate in cognition. Indeed, the SZ iNs displayed changes in neurotensin, somatostatin, and thymosin β-4 types of neuropeptides that are involved in memory functions [41–45]. Furthermore, changes in CHGB neuropeptides in the SZ iNs are noteworthy because CHGB neuropeptide forms are dysregulated in human Alzheimer’s disease (AD) brains with severe cognitive deficits [46], and CHGB neuropeptides accumulate in amyloid plaques of human AD brains [47, 48]. Thus, alterations in CHGB neuropeptides in the SZ iNs may reflect roles of CHGB neuropeptides in cognitive loss. These findings suggest that the iNs provide a model of SZ dysregulation of neuropeptides involved in cognition.

It is noted that this study assessed iNs derived from male SZ and male control subjects. Since sex differences in SZ features are known to occur [28], it will be important in future studies to compare female and male SZ and control subjects modeled by the iNs.

Overall, our findings suggest that iNs derived from SZ patients can model alterations in neuropeptide signatures that reflect those in human SZ brains. The SZ iN model of SZ will be useful in future studies for elucidating mechanisms of altered neuropeptide profiles that may participate in memory and other cognitive deficits in SZ.

## Data Availability

LC-MS/MS data files can be accessed at www.proteomexchange.org under the dataset number PXD033816, and through www.massive.ucsd.edu under the dataset number MSV000089460.
